# Exploring gender differences in medication consumption and mortality in a cohort of hypertensive patients in Northern Italy

**DOI:** 10.1186/s12889-022-13052-9

**Published:** 2022-04-15

**Authors:** David Consolazio, Maria Elena Gattoni, Antonio Giampiero Russo

**Affiliations:** 1 Epidemiology Unit, Agency for Health Protection of the Metropolitan City of Milan, Milan, MI Italy; 2 Department of Sociology and Social Research, University of Milan-Bicocca, Milan, MI Italy

**Keywords:** Gender Differences, Hypertension, Medication Consumption, Compliance, Mortality

## Abstract

**Background:**

This paper aims to assess the presence of gender differences in medication use and mortality in a cohort of patients affected exclusively by hypertension, in 193 municipalities in the Lombardy Region (Northern Italy), including Milan's metropolitan area.

**Methods:**

A retrospective cohort study was conducted (*N* = 232,507) querying administrative healthcare data and the Register of Causes of Death. Hypertensive patients (55.4% women; 44.6% men) in 2017 were identified; gender differences in medication use (treatment, 80% compliance) and deaths (from all causes and CVDs) were assessed at two-year follow-ups in logistic regression models adjusted for age class, census-based deprivation index, nationality, and pre-existing health conditions. Models stratified by age, deprivation index, and therapeutic compliance were also tested.

**Results:**

Overall, women had higher odds of being treated, but lower odds of therapeutic compliance, death from all causes, and death from CVDs. All the outcomes had clear sex differences across age classes, though not between different levels of deprivation. Comparing patients with medication adherence, women had lower odds of death from all causes than men (with a narrowing protective effect as age increased), while no gender differences emerged in non-compliant patients.

**Conclusions:**

Among hypertensive patients, gender differences in medication consumption and mortality have been found, but the extent to which these are attributable to a female socio-cultural disadvantage is questionable. The findings reached, with marked age-dependent effects in the outcomes investigated, suggest a prominent role for innate sex differences in biological susceptibility to the disease, whereby women would take advantage of the protective effects of their innate physiological characteristics, especially prior to the beginning of menopause.

**Supplementary Information:**

The online version contains supplementary material available at 10.1186/s12889-022-13052-9.

## Background

Hypertension, or high blood pressure, is a long-term medical condition in which blood pressure in the arteries is persistently elevated. Although typically asymptomatic, hypertension is one of the most important risk factors for cardiovascular diseases (CVDs), stroke, chronic kidney disease, as well as other pathological conditions [1]. Moreover, even slight elevations in arterial blood pressure are associated with significant reductions in life expectancy [2]. Besides genetic susceptibility, risk factors for hypertension include unhealthy diet (especially with high salt intake), excess body weight, physical inactivity, smoking and immoderate alcohol use [1]. Hence, like many other diseases strongly associated with lifestyle and behaviours, hypertension is unequally distributed across social groups, with higher prevalence in the less educated compared to the highly educated [3–5]. Furthermore, a pattern of inequality is also present in relation to care, with rates of awareness, treatment, and control varying according to age, education, and income levels [6–8]. Gender is another pivotal element, as hypertension prevalence and care have been reported to vary extensively between men and women [9–13], yielding contrasting results, which are strongly dependent on the context studied. Such differences are known to be driven by biological, behavioural, and socio-cultural factors. Biological factors include sex hormones and other innate sex differences that are protective against hypertension in women [12, 14]. For instance, women enjoy the protective effects of oestrogens before menopause [15], which make them less subject to salt-sensitivity [16] and contribute to a delayed onset of the disease compared to men [17]. Men and women also differ in the distribution of the aforementioned key behavioural risk factors, some of which may contribute to narrowing the gender gap in hypertension (e.g. lower physical activity in women), while others may widen the disparities outlined by biological conditions (e.g. higher smoking prevalence in men). It is important to stress that these differences in health-related behaviours are influenced by the dominant role and gender models present in society [18], so that societal and cultural values may play an important role in shaping hypertension risk exposure [19]. Beyond the socio-cultural influence on health attitudes and lifestyles, it has been reported that women are less likely to receive optimal diagnostic evaluation and therapeutic intervention than men [11, 12, 20]. Considering the relevance of the gender dimension in relation to several aspects of the disease, we aimed to explore the gender patterning of medication consumption and mortality in a cohort of subjects diagnosed exclusively with hypertension in the territory covered by the Agency for Health Protection of the Metropolitan City of Milan (ATS of Milan). As the literature suggests, the extent to which such measures vary between men and women is strictly dependent on the context studied, presumably according to different biological susceptibility, societal values and cultural norms, and healthcare provision, alongside other features of the local environment [9–19]. Hence, without context-specific knowledge about the phenomena investigated, it is not possible to develop proper interventions aimed at tackling hypertension and its related inequalities. The topic is also highly relevant in light of the epidemiological evidence concerning mortality trends in the last decade in Italy, where CVDs have been replaced by neoplasms as the primary cause of death in men, but not in women [21], likely because of the existence of gender differences in therapeutic compliance. Given the lack of such information in the territory of the ATS of Milan, this study sets out to deepen the knowledge about gender disparities in hypertension in the territory.

## Methods

### Study Population

The study was conducted querying the Administrative Healthcare Databases (AHD) of the ATS of Milan, which gathers health data for people living in the provinces of Milan and Lodi (193 municipalities), with a population of about 3.46 million people in 2017. In Italy, since 1978, the population has been fully covered by a universal and tax-funded healthcare system; in the Lombardy Region since 1997 its management has been associated with an automated system of databases, which collect a variety of information concerning services provided to beneficiaries of the healthcare system. Through 2017 AHD data we retrospectively identified the cohort of hypertensive patients aged 30–99 years (*N* = 232,507) which were not affected by any other chronic disease. Hypertensive patients were identified according to the criteria established by the 2017 Lombardy Region’s deliberation n° X/6164, which aimed to develop an innovative system to improve the assistance to patients with chronic diseases or those in vulnerable conditions. The Region provided codes to detect from the databases individuals affected by chronic conditions. Data were linked by the social security number with pharmaceutical consumption and deaths in 2018 and 2019 to assess the outcomes at two-year follow-ups. The choice to include subjects with only hypertension in the cohort was due to the possibility to account for medication use and deaths directly attributable to this specific disease, without the overlap of other medical conditions sharing the same risk profile, treatment, and outcomes.

### Measures

*Medication consumption* for each subject was derived from the Pharmaceutical Consumption Database (PCD), which encompasses information concerning all three channels of medicine supply: pharmacies, hospitals, and on behalf of the local health authorities. All the prescriptions referring to code C of the *Anatomical Therapeutic Chemical* (ATC) *classification system *[22] were included, which allowed us to identify pharmaceutical consumption related to the cardiovascular system (cardiac therapy, antihypertensives, diuretics, peripheral vasodilators, vasoprotectives, beta-blocking agents, calcium channel blockers, agents acting on the renin–angiotensin system, lipid-modifying agents). Medication use was expressed in terms of *Defined Daily Dose* (DDD), a standardized statistical measure, which allows a comparison of consumption between different products, populations, and time intervals [23]. DDD is defined as the assumed average maintenance dose per day for a medicine used for its main indication in adults. From the information available in the PCD, we extracted two complementary measures of medication intake. First, we identified as treated those who took at least one DDD of ATC code C medicines in the two-year period. Second, the continuous DDD measure (overall ATC code C consumption in the two years) was dichotomized to distinguish compliant from non-compliant patients relying on the 80% cut-off based on the early empirical definition of sufficient adherence to antihypertensive medications [24].

*Mortality* was extracted from the Register of Causes of Death (*Registro Nominativo delle Cause di Morte*, ReNCaM). Deaths from all causes and cause-specific deaths from CVDs were considered.

*Gender, age,* and *nationality* (Italian/non-Italian) were extracted from the civil registry (*Nuova Anagrafe Regionale*, NAR).

*Deprivation index* of the census block of residence was computed using data from the latest Italian census [25]. We relied on Rosano and colleagues’ index [26], which is a revised version of Caranci and colleagues’ index [27, 28]. The index was computed as the sum of the z-scores of five indicators: the percentage of individuals (15–60 y.o.) with at most primary education, the percentage of unemployed individuals (15–60 y.o.), the percentage of households who rented their homes, the percentage of single-parent families with underage children, and the average of housing crowding (number of inhabitants per 100m^2^) in each census block. The continuous score obtained was categorized in quartiles, each identifying the percentage of subjects living in very low, low, high, and high deprivation areas.

*Pre-existing health conditions* are typically measured by the presence of comorbidities, which in our case is null given the identification of a cohort of hypertensive patients with no other chronic medical conditions. Therefore, we assessed them through hospital admissions and first aid accesses in the year before the outcome considered. Data were extracted from the hospital discharges (*Schede di Dimissione Ospedaliera*) and the emergency department discharges (*Pronto Soccorso*) databases.

### Statistical Analysis

Medication use and death rates were first presented graphically, separately for women and men, and segmented by age class. Then, gender differences in the outcomes were assessed through regression models adjusted for age, nationality, deprivation index, and pre-existing health conditions. Models with deaths as outcomes were also adjusted for therapeutic compliance. Multivariable logistic regression models were used to obtain odds ratios of women versus men for each outcome. The models were also stratified by ten-year age classes and deprivation index – this last dichotomized to emphasize difference between patients living in very low/low and high/very high deprivation areas. Mortality models were additionally stratified by ten-year age classes and therapeutic compliance. Stratified mortality models were run only for subjects aged 50 years or older and were not adjusted for nationality due to insufficient sample size in some age categories. As robustness checks, we also ran models specifying a three-way interaction term between gender, age class and deprivation index (also therapeutic compliance for mortality models) to specifically account for possible gender effect modifications due to such predictors. In all models, multicollinearity was assessed. Statistical significance was set by *P*-values < 0.05 (2-sided). All analyses were conducted using Stata version 16.

## Results

**Fig.1:**
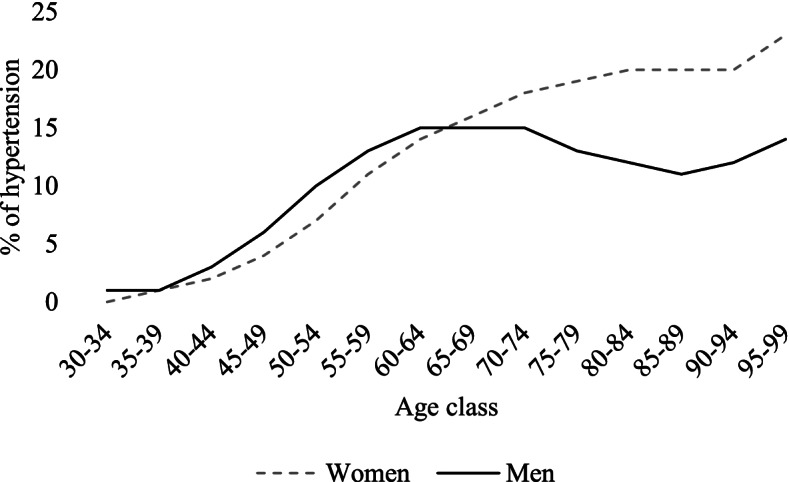
Percentage of hypertensive patients (30–99 y.o.) without any other chronic medical condition in the ATS of Milan, by gender and age class, 2017 (Women = 9.8%; Men = 8.8%; Overall = 9.4%)

**Fig. 2:**
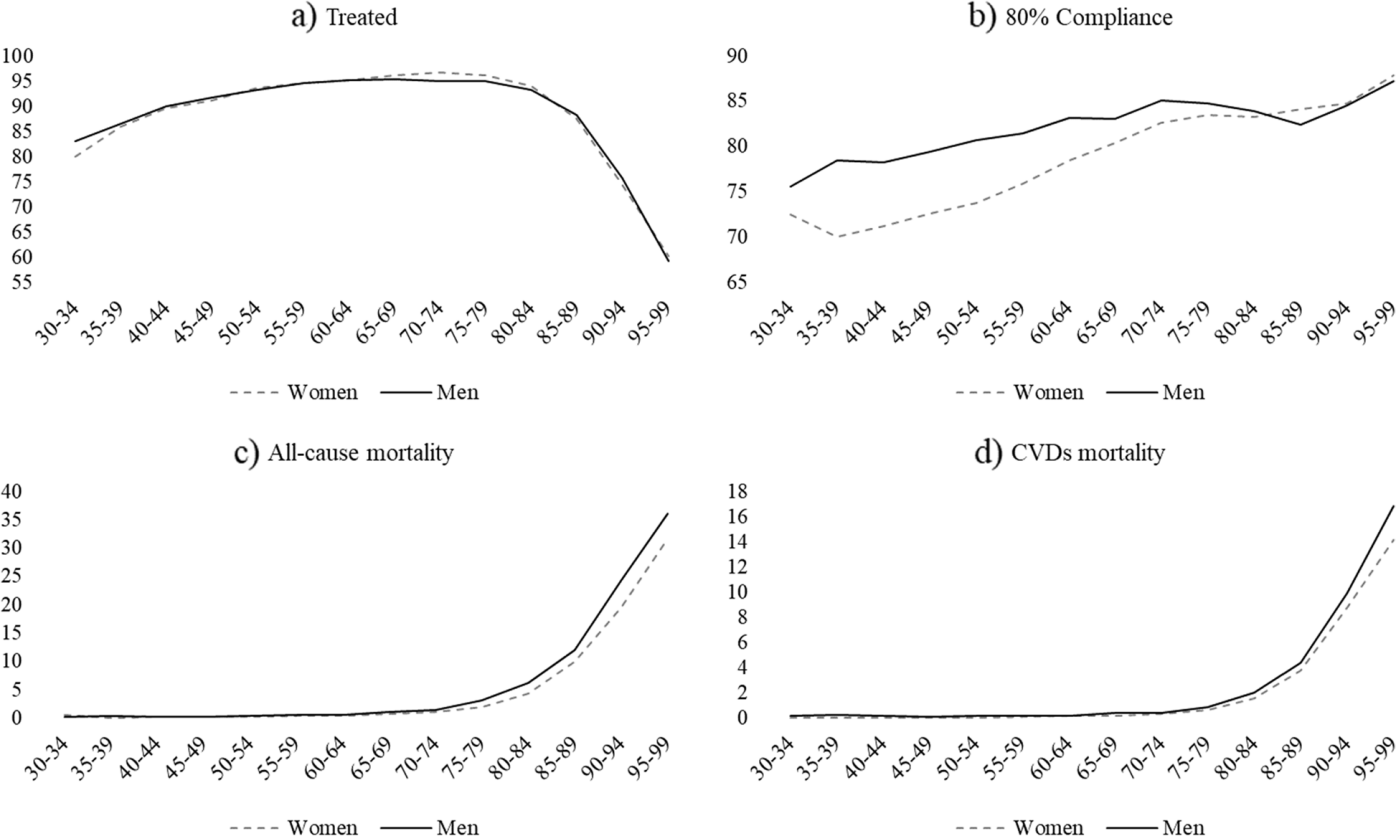
Medication consumption (percentage of treated patients, percentage of compliant patients) and deaths (all causes, CVDs) in hypertensive patients (30–99 y.o.) without any other chronic medical condition in the ATS of Milan, by gender and age class, 2018–2019

Table 1 shows descriptive statistics, overall and by gender. Figure 1 shows the percentage of subjects diagnosed with only hypertension in the territory of the ATS of Milan (the overall population for each class used as denominator was taken from the Italian National Institute of Statistics: https://demo.istat.it/). Overall, women (9.8%) have a one percentage point higher rate compared to men (8.8%), but the values are strongly dependent on age class; until the 60–64 age class rates are slightly higher in men, for whom values start to decrease in old age, slightly increasing again in the last two age classes. Conversely, women show a steady increase leading to sizeable differences compared to men in elderly age. This highlights the importance of considering age class differences in subsequent analyses on hypertension outcomes. Figure 2 provides an insight into medication use and death rates within the cohort. The percentage of treated patients slightly increases with age and starts to decrease in old age. Gender differences are present in the youngest age classes, in which women appear to be less often treated than men, and between the 60–64 and 80–84 age classes, where conversely women are treated more often than men. However, a different picture emerges when looking at the percentage of patients who reached the 80% compliance threshold in the two-year period. Women, who show lower medication adherence in the youngest age classes, gradually narrow their gap until a reversal of the pattern in old age, when their compliance is slightly higher than men’s. Concerning mortality, deaths from all causes are similar between men and women, with higher rates in men in each age class. Cause-specific deaths from CVDs follow an analogous pattern, with less pronounced gender differences, except for the oldest age class.

**Table 1:** Descriptive statistics of the cohort (*N* = 232,507)

	**N**			**%**		
	**Women**	**Men**	**Total**	**Women**	**Men**	**Total**
**Age class**						
*30–34*	374	687	1,061	0.29	0.66	0.46
*35–39*	1,038	1,723	2,761	0.81	1.66	1.19
*40–44*	2,786	4,656	7,442	2.16	4.49	3.2
*45–49*	6,045	9,022	15,067	4.69	8.7	6.48
*50–54*	10,489	13,642	24,131	8.14	13.16	10.38
*55–59*	12,583	14,842	27,425	9.77	14.32	11.8
*60–64*	14,247	14,492	28,739	11.06	13.98	12.36
*65–69*	16,625	13,888	30,513	12.9	13.4	13.12
*70–74*	16,929	11,572	28,501	13.14	11.16	12.26
*75–79*	17,931	9,596	27,527	13.92	9.26	11.84
*80–84*	14,270	5,629	19,899	11.07	5.43	8.56
*85–89*	9,663	2,795	12,458	7.5	2.7	5.36
*90–94*	4,565	936	5,501	3.54	0.9	2.37
*95–99*	1,310	172	1,482	1.02	0.17	0.64
**Nationality**						
*Italian*	121,736	99,199	220,935	94.48	95.7	95.02
*Non-Italian*	7,119	4,453	11,572	5.52	4.3	4.98
**Deprivation Index**						
*Very low*	31,419	26,715	58,134	24.38	25.77	25
*Low*	31,530	26,601	58,131	24.47	25.66	25
*High*	32,290	25,829	58,119	25.06	24.92	25
*Very high*	33,616	24,507	58,123	26.09	23.64	25
**Hospital admission**						
*No*	106,805	85,157	191,962	82.89	82.16	82.56
Yes	22,050	18,495	40,545	17.11	17.84	17.44
**First-aid access**						
*No*	82,081	69,063	151,144	63.7	66.63	65.01
*Yes*	46,774	34,589	81,363	36.3	33.37	34.99
**Treated**						
*No*	8,756	6,709	15,465	6.8	6.47	6.65
*Yes*	120,099	96,943	217,042	93.2	93.53	93.35
**80% Compliance**						
*No*	25,702	18,352	44,054	19.95	17.71	18.95
*Yes*	103,153	85,300	188,453	80.05	82.29	81.05
**Death (all causes)**						
*No*	125,232	101,849	227,081	97.19	98.26	97.67
*Yes*	3,623	1,803	5,426	2.81	1.74	2.33
**Death (CVDs)**						
*No*	127,464	103,037	230,501	98.92	99.41	99.14
*Yes*	1,391	615	2,006	1.08	0.59	0.86

Table 2 reports the results of the multivariable regression models. Adjusting for confounders, women in the cohort have statistically significantly higher odds of being treated compared to men (OR = 1.11; 95% C.I. = 1.07–1.15), though the picture changes radically when considering the volume of pharmaceutical consumption, in relation to which women are less likely than men to reach the 80% compliance threshold (OR = 0.79; 95% C.I. = 0.77–0.80). However, despite a lower medication use, women also show lower odds of death from both all causes (OR = 0.77; 95% C.I. = 0.72–0.82) and from CVDs (OR = 0.80; 95% C.I. = 0.72–0.89) compared to men.

**Table 2:** Regression models of drug consumption and mortality in the cohort (N = 232,507)

	**Treated**		**80% Compliance**		**All-cause mortality**		**CVDs mortality**	
	**Odds Ratio**	**95% C.I**	**Odds Ratio**	**95% C.I**	**Odds Ratio**	**95% C.I**	**Odds Ratio**	**95% C.I**
**Gender**								
*Men (ref.)*	1	-	1	-	1	-	1	-
*Women*	1.11	[1.07; 1.15]	0.79	[0.77; 0.80]	0.77	[0.72; 0.82]	0.80	[0.73; 0.89]
**Deprivation Index**								
*Very Low (ref.)*	1	-	1	-	1	-	1	-
*Medium Low*	0.99	[0.94; 1.04]	1.04	[1.01; 1.07]	1.08	[0.99; 1.18]	1.01	[0.89; 1.16]
*Medium High*	0.92	[0.88; 0.97]	1.04	[1.01; 1.07]	1.18	[1.08; 1.28]	1.12	[0.98; 1.27]
*Very High*	0.90	[0.86; 0.94]	1.06	[1.03; 1.09]	1.24	[1.14; 1.35]	1.15	[1.02; 1.31]
**Age class**								
*30–34 (ref.)*	1	-	1	-	1	-	1	-
*35–39*	1.43	[1.18; 1.73]	1.05	[0.89; 1.24]	0.79	[0.20; 3.19]	1.57	[0.18; 14.10]
*40–44*	1.99	[1.67; 2.37]	1.07	[0.92; 1.24]	0.70	[0.20; 2.46]	1.08	[0.13; 8.78]
*45–49*	2.37	[2.00; 2.80]	1.14	[0.99; 1.31]	0.63	[0.19; 2.11]	0.54	[0.07; 4.44]
*50–54*	3.14	[2.66; 3.71]	1.21	[1.05; 1.40]	1.05	[0.33; 3.35]	1.11	[0.15; 8.21]
*55–59*	3.76	[3.18; 4.44]	1.31	[1.13; 1.50]	1.45	[0.46; 4.58]	1.62	[0.22; 11.82]
*60–64*	4.11	[3.48; 4.86]	1.49	[1.29; 1.71]	1.84	[0.58; 5.81]	1.75	[0.24; 12.74]
*65–69*	4.70	[3.97; 5.57]	1.58	[1.37; 1.82]	2.88	[0.92; 9.04]	2.56	[0.35; 18.48]
*70–74*	4.68	[3.95; 5.54]	1.84	[1.59; 2.12]	3.99	[1.28; 12.51]	3.48	[0.48; 25.04]
*75–79*	4.22	[3.56; 5.00]	1.90	[1.65; 2.19]	7.06	[2.26; 22.06]	6.63	[0.93; 47.46]
*80–84*	2.73	[2.30; 3.23]	1.87	[1.62; 2.16]	13.97	[4.47; 43.65]	15.58	[2.18; 111.36]
*85–89*	1.24	[1.05; 1.47]	1.93	[1.67; 2.24]	32.71	[10.47; 102.14]	36.13	[5.06; 258.03]
*90–94*	0.50	[0.42; 0.59]	2.10	[1.79; 2.46]	79.50	[25.44; 248.44]	88.73	[12.42; 633.90]
*95–99*	0.26	[0.21; 0.32]	2.77	[2.25; 3.41]	198.86	[63.34; 624.31]	170.33	[23.76; 1,221.25]
**Citizenship**								
*Italian (ref.)*	1	-	1	-	1	-	1	-
*Other*	0.59	[0.56; 0.63]	0.96	[0.92; 1.01]	0.77	[0.56; 1.04]	0.87	[0.55; 1.39]
**Hospital admission**								
*No (ref.)*	1	-	1	-	1	-	1	-
*Yes*	1.50	[1.42; 1.59]	1.07	[1.04; 1.10]	5.26	[4.92; 5.62]	2.65	[2.39; 2.94]
**First-aid access**								
*No (ref.)*	1	-	1	-	1	-	1	-
*Yes*	1.74	[1.67; 1.81]	0.97	[0.95; 0.99]	2.34	[2.18; 2.52]	1.94	[1.74; 2.17]
**80% Compliance**								
*No (ref.)*	N.A		N.A		1	-	1	-
*Yes*					1.39	[1.27; 1.51]	1.55	[1.34; 1.78]

**Fig. 3:**
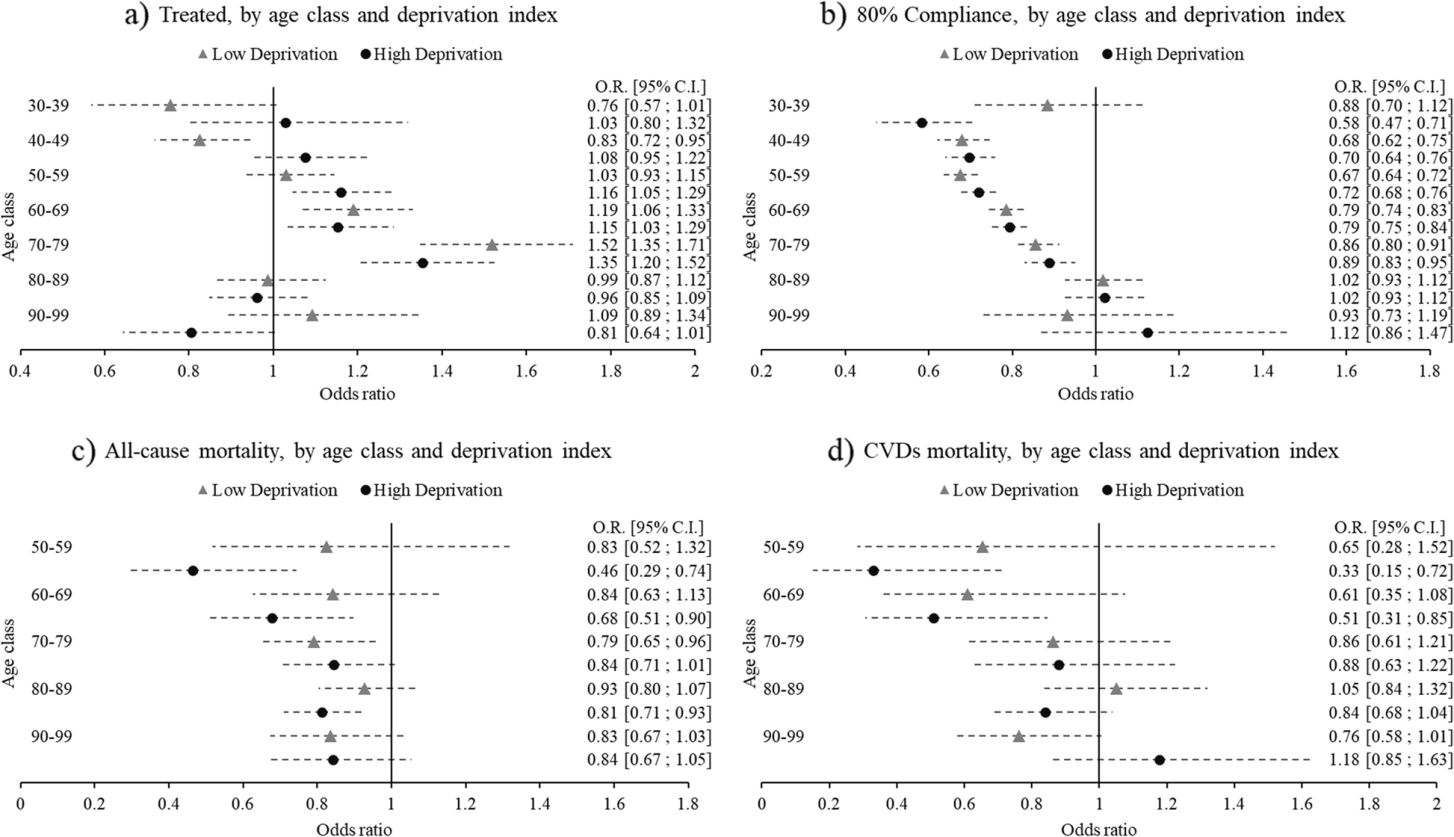
Women vs Men (ref.) odds of (**a**) being treated; (**b**) therapeutic compliance; (**c**) deaths from all causes; (**d**) deaths from CVDs, stratified by ten-year age class and deprivation index (very low/low – high/very high), adjusted for nationality, hospital admissions, first-aid access in the previous years (a, b, c, d) and therapeutic compliance (only c and d)

**Fig. 4:**
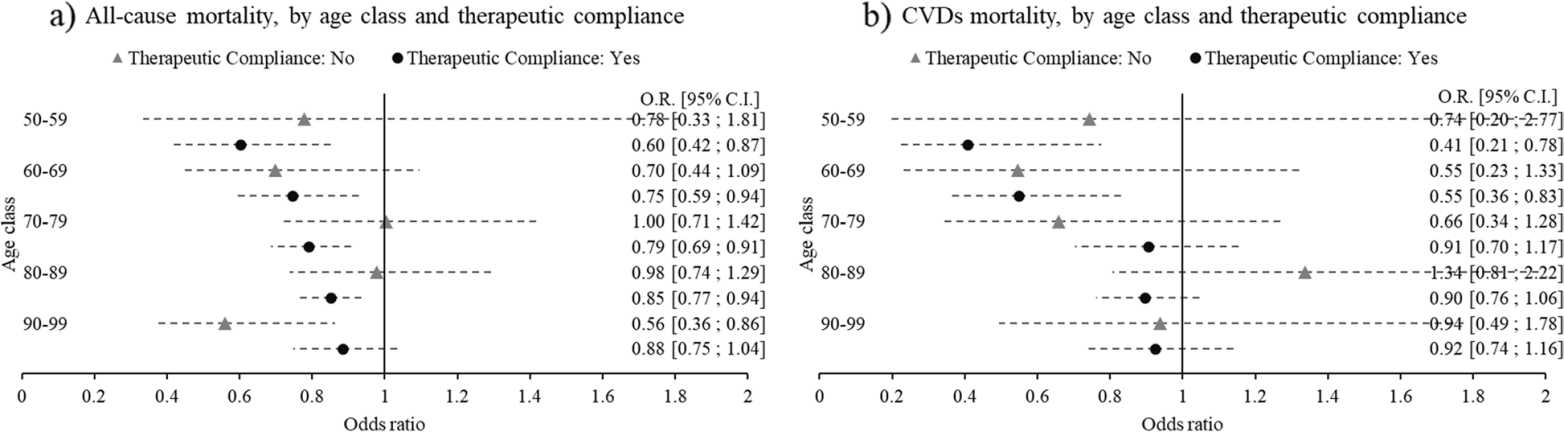
Women vs Men (ref.) odds of (**a**) deaths from all causes; (**b**) deaths from CVDs, stratified by ten-year age class and therapeutic compliance index (very low/low – high/very high), adjusted for deprivation index, hospital admissions, and first-aid access in the previous years

A quite different picture emerges when age (ten-year classes) and deprivation index (dichotomous: low/very low; high/very high) are included as modifiers, and not simply as confounders. Figure 3 shows the results of age and deprivation stratified models in forest plots, where women versus men odds ratios are presented. Values greater than 1 (at the right of the vertical line) imply higher odds for women compared to men in relation to the outcome of interest; non-overlapping 95% C.I. bars imply a statistically significant difference. The odds of being treated are systematically higher in women compared to men only in the 60–69 and 70–79 age classes (Fig. 3a). Concerning therapeutic compliance, compared to men, women are less likely to reach adherence in the youngest classes, with reducing differences as age increases, disappearing altogether in the last two age classes (Fig. 3b). Regarding mortality, the most evident results are the lower odds of death from all causes (Fig. 3c) and from CVDs (Fig. 3d) in women in the 50–59 and 60–69 age classes living in high deprivation areas, compared to men living in similar contexts. However, in relation to deaths – especially for the latter – the emergence of a pattern is influenced by the low deaths rates at two-year follow-ups. Overall, none of the four outcomes examined demonstrates a relevant pattern of variation according to deprivation index. Finally, according to models stratified by age class and therapeutic compliance, women in adherence with their therapy have significantly lower odds of deaths from all causes compared to their male counterparts, a protective effect that diminishes when moving from the youngest to the oldest age classes (Fig. 4a). A similar pattern is present in relation to death from CVDs, though weakened again by low sample size (Fig. 4b). Models implemented with a three-way interaction term as robustness checks achieved findings analogous to those that emerged from stratified models (Figures A1-A6 in the Additional File). Results of these models were presented graphically as adjusted predictions at the means, expressing, for each outcome, the predicted probability for each combination of the categories involved in the interaction terms, at the means of (i.e. keeping constant) the other covariates. As visible from the graphs, the results are in line with those that emerged from the respective stratified models, including the non-significance of the deprivation index in shaping medication consumption and deaths differently between sexes and across age classes, notable from the similarity between the low and high deprivation curves in each graph.

## Discussion

In this study, we investigated the presence of gender patterning in medication use and deaths in a cohort of patients diagnosed exclusively with hypertension in the territory covered by the ATS of Milan. The use of the term *gender* rather than *sex* was an explicit choice to frame differences in the pathology that go beyond the purely biological perspective. Indeed, the concept of gender involves culturally and socially determined roles, conventions, and behaviours that contribute to shaping relations between males and females, especially in terms of power, prestige, discrimination, and access to resources, due to socially constructed models of masculinity, which result in women bearing the major burden of negative health effects from gender-based social hierarchies [29]. Distinguishing between sex and gender differences in health outcomes is crucial to conceive specific interventions to promote public health. Sex differences originate from biological processes, thus being somehow ‘unavoidable’ and ‘acceptable’ [30] and to be treated as a purely medical condition. On the contrary, as gender differences are rooted in the social environment they should be considered *inequities*, which are by definition ‘avoidable’ and ‘unnecessary’ [30], and addressed by means of broader interventions focusing on their social patterning. As social, cultural and economic factors play an important role in shaping diagnosis and subsequent treatment[31], we additionally investigated the effect of a socioeconomic status’ (SES) proxy on hypertension-related outcomes. This allowed us to check for the possible existence of both gender and socioeconomic inequalities in hypertension-related outcomes. Moreover, gender and SES might also act together in shaping risk profiles as, according to intersectionality theory, the combination of different social categories may unveil interacting mechanisms that noticeably differ from the isolated effects of the same categories [5, 32, 33]. On the one hand, it is known that socioeconomic inequalities do not necessarily affect men and women to the same extent [32, 34, 35]; on the other hand, as regards the specific pathology studied, women enjoy the protective effects of oestrogens before the beginning of menopause [9–13], so that this and other innate physiological features [14] might mitigate the presence of stronger SES effects compared to men. Such favourable biological endowment is tangible from the examination of the pathology’s rates according to age class, whereby after menopause onset age (which is on average 51) women’s rates exceed those of men, and the gap widens with age. Specifically, women’s rates are lower than men’s until the 60–64 age class, consistent with literature indicating that development of hypertension may not occur until 5 to 10 years after the beginning of menopause [36, 37]. In line with this, women’s lower pharmaceutical consumption detected in the study might be a consequence of a less pronounced need, as the lower therapeutic adherence compared to men would otherwise go against the evidence of higher chances of being treated and of lower death rates. Indeed, a Swedish study conducted with administrative healthcare data reported that women are less likely to be prescribed antihypertensive medicines than men, especially at a young age, with reducing differences as age increases [38], supporting the hypothesis of a lower consumption as a consequence of a lower need. As regards SES, although overall the deprivation index was associated with medication use and mortality, there appeared to be no gender differences in relation to its effects. Socially disadvantaged individuals were less likely to be treated but had higher therapeutic adherence and mortality compared to the better off. Thus, among the treated, the disadvantaged might have on average higher medication use because of worse health conditions, as also suggested by the notable deprivation gradient in all-cause mortality. Overall, the results reached suggest that in Milan’s metropolitan area differences between women and men in hypertension outcomes – which appeared to be markedly age-dependent – would be chiefly attributable to biological features, with less space for socioeconomic and cultural conditions. This implies that, concerning the specific case examined, such differences are not conceivable as *inequities*, being mostly the product of natural sex-related characteristics. Such findings are not novel within the literature, which has reported mixed findings. Though some studies have identified a female disadvantage in hypertension treatment [39], CVDs treatment [40, 41], and in being in target with risk factor management [42, 43], others found opposing [44–47] or contrasting results [5, 48–50]. Hence, gender differences in hypertension treatment stand out for being a context- and age-dependent phenomenon. Concerning the context studied, leveraging socioeconomic factors would be of paramount importance to tackle social inequalities in hypertension-related outcomes, while there is no evidence for the need to focus on the gender dimension, in its sociological meaning, as the differences that emerged between men and women were likely to be dependent on their distinct physiology in relation to blood pressure risk and control [14].

This study is subject to some limitations that should be noted. Firstly, our data refers to patients diagnosed exclusively with hypertension and without any other complications. On the one hand, this represents a limitation as we necessarily focused on a minority of all patients with hypertension in the study area. On the other hand, this was an explicit choice to enable us to assess the association between the outcomes investigated and the treatment without incurring bias. This would not have been possible if including subjects with comorbidities, as ATC code C medications are not a prerogative of hypertension treatment. Secondly, we are aware of several factors influencing medication use that have not been included in the models. Among these, individual SES is known to play a pivotal role in shaping patient compliance [51–54], but having no information about this, we relied on an aggregate area deprivation measure. Though it can be considered a proxy, caution is needed when interpreting this measure, to avoid the risk of incurring the *ecological fallacy *[55]. However, despite their limitations, area-based measures of deprivation have been shown to act as a good proxy of individual SES when considered in relation to health outcomes [56–58]. Another factor is biologic susceptibility, which is typically measured through family history with the disease. Unfortunately, such information was not available in the administrative data source. Thirdly, we were able to assess treatment, but we had no means to check whether therapeutic control was reached, due to the unavailability of systolic and diastolic blood pressure measurements. Fourthly, armed with information exclusively about medication consumption – and not pharmaceutical prescriptions – we could only measure therapeutic adherence based on the assumption that chronic patients need at least one DDD of ATC code C medicines per day, hence compliance was computed on a common 730 (365 × 2) days denominator, inevitably disregarding the different therapies prescribed to each subject in the cohort. Moreover, recent studies have questioned the practice of relying on the 80% threshold as a general standard for therapeutic compliance [59–62]. Finally, we need to consider the possibility of selection bias in the identification of the cohort, as excluding subjects with comorbidities may have led us to perform the analysis on a sample with different baseline characteristics compared to the overall set of hypertensive patients in the study area.

## Conclusions

Clear differences emerged in relation to medication use and mortality between women and men but attributing them to innate sex differences in biological susceptibility to hypertension seems to be more plausible than relying on a socio-cultural explanation. However, this study constitutes only a first step to explore the existence of gender inequalities in pharmaceutical consumption for CVDs. Specific information on prescriptions and adherence, as well as ad-hoc survey data aiming at inquiring the impact of socioeconomic conditions on the management of chronic diseases, would improve knowledge about the role of the gender dimension in shaping treatment and control.

## Supplementary Information

**Additional file 1:** **Figure A1. **Adjustedpredictions (with 95% C.I.) of being treated, age*gender*deprivation interaction, at the means of nationalityand pre-existing health conditions. **Figure A2. **Adjusted predictions (with 95% C.I.) of being in therapeutic compliance, age*gender*deprivation interaction, at the means of nationality andpre-existing health conditions. **Figure A3. **Adjusted predictions (with95% C.I.) of death from all causes, age*gender*deprivation interaction, at themeans of pre-existing health conditions and therapeutic compliance. **Figure A4. **Adjusted predictions (with 95% C.I.) of death from CVDs, age*gender*deprivation interaction, at the means of pre-existing health conditions and therapeutic compliance. **Figure A5. **Adjusted predictions (with 95% C.I.) of death from all causes, age*gender*compliance interaction, at the means of deprivation index and pre-existing health conditions. **Figure A6. **Adjusted predictions (with95% C.I.) of death from CVDs, age*gender*compliance interaction, at the means of deprivation index and pre-existing health conditions.

## Data Availability

The dataset generated and analysed during the current study is not publicly available due to privacy concerns, as it originates from administrative healthcare databases, which are subject to privacy restrictions.

## Abbreviations

AHD: Administrative Healthcare Databases
ATS of Milan: Agency for Health Protection of the Metropolitan City of Milan
ATC: *Anatomical Therapeutic Chemical*
CVDs: Cardiovascular diseases
DDD: *Defined Daily Dose*
PCD: Pharmaceutical Consumption Database
SES: Socioeconomic Status
