# Supplementary material for: Exploring gender differences in medication consumption and mortality in a cohort of hypertensive patients in Northern Italy
Source: BMC Public Health. 2022 Apr 15;22:768. doi: 10.1186/s12889-022-13052-9 (PMC9013154; doi:10.1186/s12889-022-13052-9)
Supplement: Supplementary file 1 — Additional file 1: Figure A1. Adjustedpredictions (with 95% C.I.) of being treated, age*gender*deprivation interaction, at the means of nationalityand pre-existing health conditions. Figure A2. Adjusted predictions (with 95% C.I.) of being in therapeutic compliance, age*gender*deprivation interaction, at the means of nationality andpre-existing health conditions. Figure A3. Adjusted predictions (with95% C.I.) of death from all causes, age*gender*deprivation interaction, at themeans of pre-existing health conditions and therapeutic compliance. Figure A4. Adjusted predictions (with 95% C.I.) of death from CVDs, age*gender*deprivation interaction, at the means of pre-existing health conditions and therapeutic compliance. Figure A5. Adjusted predictions (with 95% C.I.) of death from all causes, age*gender*compliance interaction, at the means of deprivation index and pre-existing health conditions. Figure A6. Adjusted predictions (with95% C.I.) of death from CVDs, age*gender*compliance interaction, at the means of deprivation index and pre-existing health conditions. [file 12889_2022_13052_MOESM1_ESM.docx]

## **Data Supplement**

*Figure A1: Adjusted predictions (with 95% C.I.) of being treated, age*gender*deprivation interaction, at the means of nationality and pre-existing health conditions.*


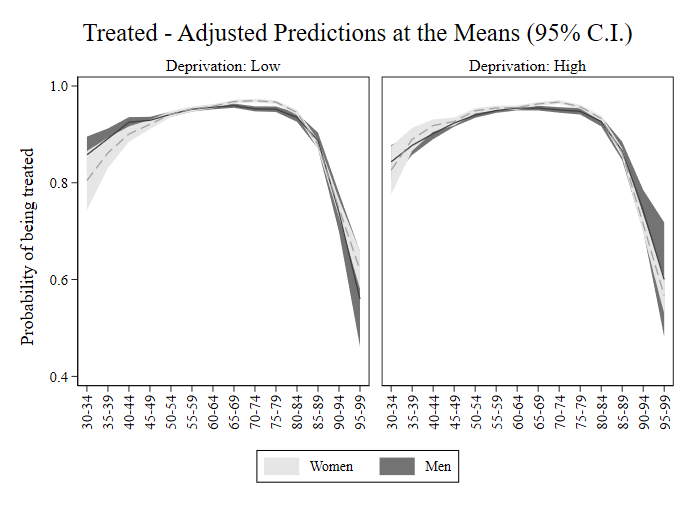


*Figure A2: Adjusted predictions (with 95% C.I.) of being in therapeutic compliance, age*gender*deprivation interaction, at the means of nationality and pre-existing health conditions.*


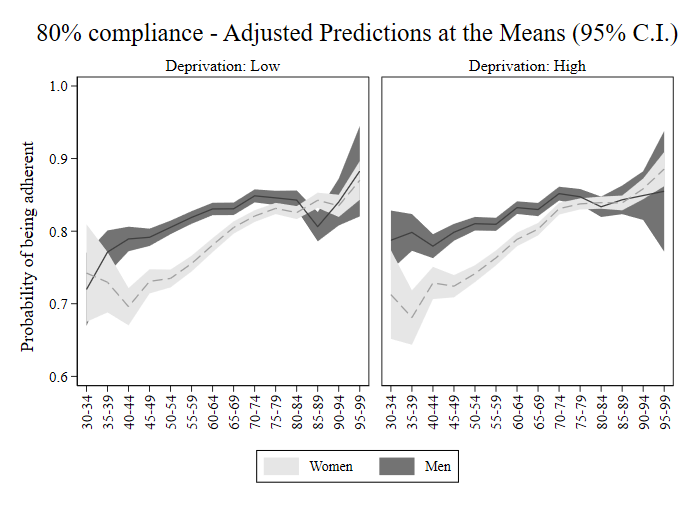


*Figure A3: Adjusted predictions (with 95% C.I.) of death from all causes, age*gender*deprivation interaction, at the means of pre-existing health conditions and therapeutic compliance.*


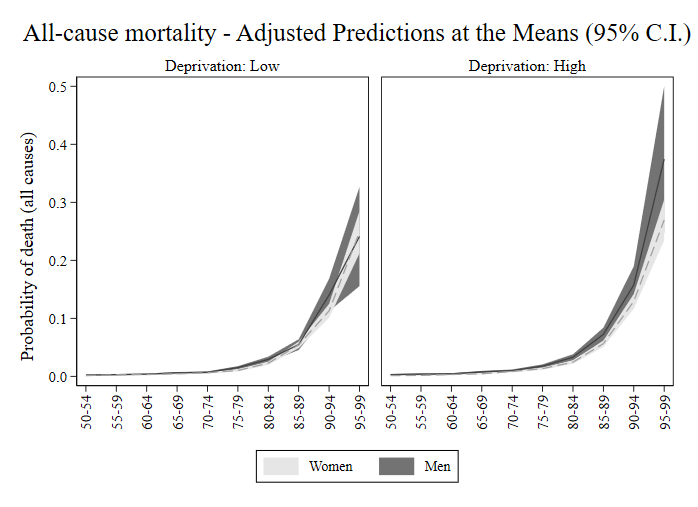


*Figure A4: Adjusted predictions (with 95% C.I.) of death from CVDs, age*gender*deprivation interaction, at the means of pre-existing health conditions and therapeutic compliance.*


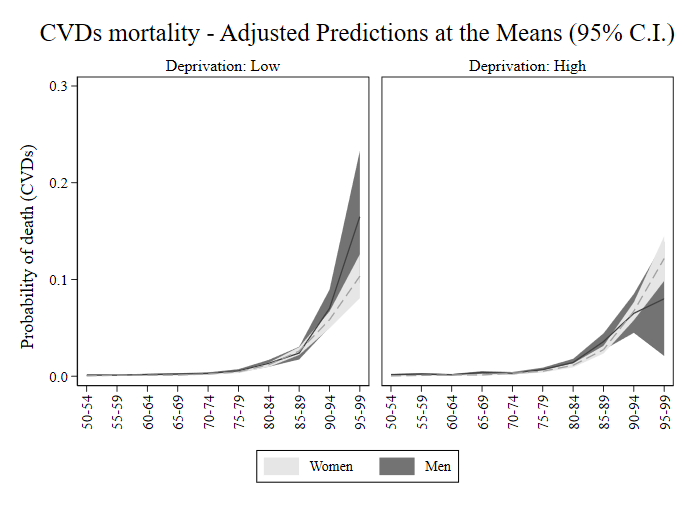


*Figure A5: Adjusted predictions (with 95% C.I.) of death from all causes, age*gender*compliance interaction, at the means of deprivation index and pre-existing health conditions.*

*
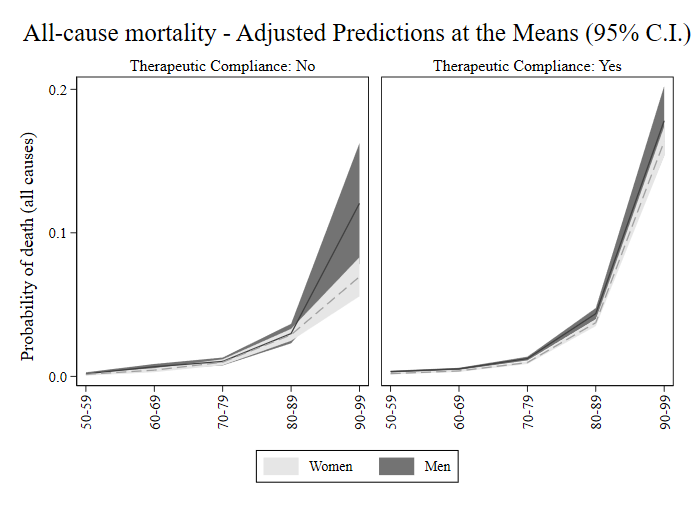
*

*Figure A6: Adjusted predictions (with 95% C.I.) of death from CVDs, age*gender*compliance interaction, at the means of deprivation index and pre-existing health conditions.*

*
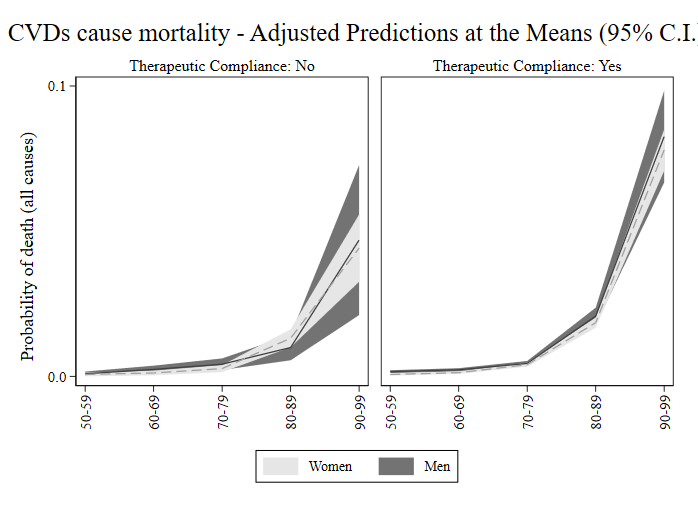
*
